# A newly-recorded species, *Roeslerstammia erxlebella* (Fabricius, 1787) (Lepidoptera, Roeslerstammiidae) from Korea, with a key to species of the genus and DNA barcoding analysis

**DOI:** 10.3897/BDJ.13.e171683

**Published:** 2025-11-05

**Authors:** Jinsung Park, Dae-kyeong Ra, Sora Kim

**Affiliations:** 1 Jeonbuk National University, Jeonju, Republic of Korea Jeonbuk National University Jeonju Republic of Korea

**Keywords:** taxonomy, morphology, taxonomic review, Gracillarioidea, identification key, CO1 gene, DNA Barcode

## Abstract

**Background:**

The genus *Roeslerstammia* Zeller, 1839, the type genus of the family Roeslerstammiidae, comprises a total of four species on a global scale. The type species, *Roeslerstammia erxlebella* (Fabricius, 1787) is distributed across the Palaearctic Region, from Europe to Japan. However, the presence of this species has only been confirmed in European countries, Russia and Japan.

**New information:**

The present study reports the first record of *Roeslerstammia erxlebella* in Korea, specifically from Odae-san National Park. This paper constitutes a review of the taxonomic history of the family Roeslerstammiidae and the genus *Roeslerstammia*. A thorough taxonomic account of the recently documented species, *R. erxlebella*, is presented, accompanied by a taxonomic key and an illustrated map delineating the geographical distribution of the genus *Roeslerstammia*. Furthemore, the DNA Barcode data of a Korean individual was made available, alongside public data from BOLD systems. The DNA Barcoding analysis further indicates that the Korean individual is *R. erxlebella*.

## Introduction

The family Roeslerstammiidae is widely distributed across the Palaearctic, Oriental and Australian Regions, with the highest diversity in the Australian Region (Hirowatari et al. 2017). Globally, the family Roeslerstammiidae is reported as 13 genera and 57 species (van Nieukerken 2011, Hirowatari et al. 2017) and the majority of its larvae exhibit leaf-mining behaviour (Bontsema et al. 2017). The validity of the family name, Roeslerstammiidae, has been a subject of debate (Heppner 2005, van Nieukerken and Karsholt 2006). The debate arises because the family Roeslerstammiidae was originally erected by Bruand (1851) as Roslerstammiidae, containing a misspelling and lacking the type species *R. erxlebella*. However, the spelling was corrected under ICZN 32.5.3 (Handlirsch 1925) and *R. erxlebella* had already been included in the genus by Zeller (1839). Furthermore, *R. erxlebella* was designated as the type species by Fletcher (1929). Therefore, Roeslerstammiidae remains the valid family name.

Based on the morphology, the systematic position of the family Roeslerstammiidae is primarily defined by the characteristics of venation, genitalia and antennae (Kyrki 1983, Robinson 1988, Davis and Robinson 1998). Regarding venation, the R5 venation of the fore-wing terminates in the costa and the hind-wing CuA1 venation and M3 venation originate from a single point. Male genitalia possess valvae with spine-like saccular processes and female genitalia exhibit a linear signum. The dorsal scale of Flagellomeres consists of two rows (Davis and Robinson 1998). Based on morphological evidence, the family Roeslerstammiidae is included in the superfamily Gracillarioidea. The molecular phylogenetic studies also supported this classification. In the molecular phylogenetic study of Gracillarioidea (Kawahara et al. 2011), the family Roeslerstammiidae is identified as the sister group to Gracillariidae and Yponomeutidae within the G.B.R.Y. clade.

The *Roeslerstammia* species are reported to have only four species globally: *R. erxlebella* (Fabricius, 1787), *R. pronubella* (Denis and Schiffermüller 1775), *R. metaplastica Meyrick 1921* and *R. tianpingshana* Hirowatari et al. 2017. The newly-recorded species *Roeslerstammia erxlebella* is the type species of the genus *Roeslerstammia* and is distributed across the Palaearctic Region, from Europe to Japan (Hirowatari et al. 2017). According to the faunistic data of Europe (Karsholt 2001), *R. erxlebella* is present in most European countries. In Russia and Japan, this species was previously described as *R. durulguensis* and *R. bella*, respectively, but these names have since been synonymised. In Korea, a single species, *R. nitidella*, was recorded in 1983 (Shin et al. 1983). This was the first and only record of the family Roeslerstammiidae and the genus *Roeslerstammia* from Korea. This species was later synonymised with *R. pronubella*. This study provides taxonomic accounts of the species with detailed morphological illustrations. Additionally, we give a comprehensive checklist and a key to the species of the genus *Roeslerstammia*.

## Materials and methods

**Specimen collection and preparation**

For this study, specimens were collected using a light trap, equipped with a mercury vapour lamp (220V/400W) from dusk until midnight. The moths were euthanised with a 30% ammonia solution and stored in a freezer. They underwent a softening treatment in a sealed container at 50°C with high humidity for 30 minutes to facilitate specimen handling. Afterwards, their wings were spread and left to dry in a drying oven at 50°C for a month.

**Dissection and identification**

Abdominal dissections were performed following a modified version of the method originally described by Robinson (1976), using an EZ4 stereomicroscope (Leica Microsystems, Germany). The specimens and dissected genitalia were examined with a Canon EOS 6D camera, equipped with a Canon Macro Lens EF 100 mm, mounted on a Stack Shot Macro Package and illuminated by a Leica LED 5000 HDI dome light. Alternatively, a Tucsen Dhyana 400 DC digital camera was used in combination with a Leica S8AP0 stereomicroscope and the same dome illuminator. Image acquisition and processing were carried out using Helicon Focus software (version 8.2.2 Pro, Helicon Soft, Ukraine) or Mosaic software (version 2.4) in conjunction with Adobe Photoshop 2022 (version 23.5.1, Adobe, USA). Additionally, high-resolution images of the genitalia were obtained with a Leica Z16 APO stereomicroscope (Leica Microsystems, Germany) using Optview software (Korea Lab Tech, Republic of Korea).

**DNA extraction, polymerase chain reaction (PCR) and sequencing**

DNA extraction was implemented using DNeasy Blood and Tissue kits (Quiagen, Hilden, Germany), based on the manufacturer's protocol. DNA quantification was calculated by Qubit 4 (ThermoFisher Scientific, Waltham, Massachusetts, USA). Polymerase chain reaction (PCR) amplified the Lepidopteran DNA Barcode region, 612 bp of the 5' region of Cytochrome Oxidase 1 (COI) gene (Hebert et al. 2003, Hebert et al. 2004, Hajibabaei et al. 2006, Prado et al. 2011). The forward primer LepiF, 5'-ATTCAACCAATCATAAAGATAT-3' and the reverse primer EnhLepR 5'-CTCCWCCAGCAGGATCAAAA-3' were used for amplification. The thermocycling programme consisted of 95℃ for 2 min for initial denaturation, followed by 40 cycles of denaturation at 95℃ for 30 s, annealing at 55℃ for 30 s, extension at 72°C for 1 min and a final extension at 72°C for 5 min. PCR products were checked in 1.2% agarose gels and purified by QIAquick PCR purification kit (QIAGEN, Hilden, Germany) following the manufacturer's protocol. Purified samples were sequenced at Macrogen (Seoul, Republic of Korea).

**DNA barcoding analysis**

A total of 38 sequences were analysed, including the collected sample's sequence (GenBank accession number: PX249788). The rest of the sequences were downloaded from the Barcode of Life Data Systems (BOLD Systems, BINs: AAE7694, AAX8824, ABV0137). The sequence alignment and DNA barcoding analysis were conducted using the following: Mafft ver. 7 (Katoh and Standley 2013), BioEdit ver. 7. 7 (Hall 1999) and Mega ver. 11 (Tamura et al. 2021). The Neighbour-Joining-tree (NJ-tree) construction and the pairwise distance of the dataset were calculated, based on the Kimura 2-parameter model.

**Distribution map**

The distribution map was made using QGIS Version 3.34.10 (QGIS Development Team, Zurich, Switzerland) for Windows open source.

## Taxon treatments

### Roeslerstammia erxlebella

(Fabricius, 1787)

2E83DDD3-B044-5FDF-A66F-FCB842441F3D

*Alucita erxlebella* Fabricius, 1787 - Fabricius (1787): 256. Type locality: Goettinga (Germany)

#### Materials

- **Type status:** Other material. **Occurrence:** individualCount: 1; sex: male; lifeStage: adult; occurrenceID: AB684BF0-ECC0-5F98-9969-7612F807D760; **Taxon:** scientificName: Roeslerstammia erxlebella; kingdom: Animalia; phylum: Arthropoda; class: Insecta; order: Lepidoptera; family: Roeslerstammiidae; genus: Roeslerstammia; specificEpithet: erxlebella; taxonRank: Species; **Location:** country: Korea; countryCode: 82; stateProvince: Kangwon; county: Jinbu-myeon; locality: Odae-san National Park, Mt. Odae-san; verbatimElevation: 767 m; verbatimCoordinates: 37°45'57"N 128°34'39"E; decimalLatitude: 37.7658; decimalLongitude: 128.5775; **Event:** samplingProtocol: Light trap; eventDate: 13/7/2024

#### Description

**Adult** (*Fig. 1*). Wing span 15 mm. Head frons covered with yellowish-brown scales, smooth; vertex vestiture rough, yellowish-brown dorsally, brown cranially; Ocellus absent; antennae slightly shorter than fore-wing; scape yellowish-brown, approximately 1.5 times thicker than flagellum, cylindirical, ventro-anterior scales rough, thin, hair-like; flagellum approximately 50 segments, ciliated ventrally, divided into four portions with distinct dorsal colours; ratio of portion approximately 2:1:1:1; basal first portion goldish-brown, tinged with brown and pulplish scales; second portion dark brown; third portion whitish; fourth portion dark brown; proboscis naked; labial palpus cylindrical, equal thickness across all segments, apically tipped, yellowish-brown; second segment more than twice length of first segment; third segment slightly longer than second segment. Thorax goldish-brown, tinged with pulplish scales. Abdomen brown. Fore-wing metallic, goldish-brown without streaks, tinged with pulplish scales around base. Hind-wing dark brown, scattered with yellowish scales around base.

Male genitalia (Fig. 2). Uncus symmetrical, concave anteriorly, fused with tegumen basally, trapezoidal basally, bilobed apically, sparsely setose on two lobes. Gnathos well developed with thin two arms; two arms curved posteriorly, united medially with membranous part. Tegumen broad, slightly broader than valva, ring-shaped; discoidal pores densely distributed. Vinculum fused with saccus. Saccus thumb-shaped, rounded anteriorly, approximately one-fifth length of valva. Valva slightly longer than length of uncus to saccus, widest at two-thirds from base, hairy, spatulate end. Sacculus broad basally, narrowed distally, hairy, extended until two-thirds of valva; saccular process present apically, tapering. Phallus robust, slightly curved.

#### Diagnosis

*Roeslerstammia erxlebella* is morphologically similar to *R. pronubella*, sharing the goldish-brown ground colour of fore-wings. The genitalic features are also similar by having the extended Sacculus with the terminal saccular process. However, the two species can be distinguished by the detailed genitalic characters. In the male genitalia, *R. erxlebella* possesses a relatively more pointed apex of Uncus and the width of valva is slightly narrower from the saccular process to the apex, resulting in a hand-shaped apex, whereas, *R. pronubella* has a more rounded and circular apex of valva. The phallus of *R. erxlebella* is curved. In contrast, R. pronubella has an approximately straight phallus and the phallus is basally angled. In the female genitalia, *R. erxlebella* has a rather larger corpus bursa. Additionally, the corpus bursa and the ductus bursae are more easily distinguished compared to *R. pronubella*. The most notable difference between the two species is the presence of signum. The cross-shaped signum is present in R. erxlebella and the signum is absent in *R. pronubella* (Hirowatari et al. 2012).

#### Distribution

Korea (this study), Europe (Great Britain, from France to Ukraine and South Fennoscandia to the Urals), Russia, Japan (Karsholt 2001, Kishida 2011, Lelej 2016) (Fig. 3).

#### Ecology

The host plants of this species are *Tilia* sp., *Corylus* sp., *Acer* sp. and *Betula* sp. (Hirowatari et al. 2012).

#### Notes

The shape of the uncus is often used as a diagnostic character in this genus (Hirowatari et al. 2012, Hirowatari et al. 2017). However, a rigorous examination of literature (Kyrki 1983, Heppner 2005, Hirowatari et al. 2012, Bontsema et al. 2017, British Lepidoptera 2018, Lepidoptera Dissection Group UK 2025) demonstrates that the shape of the uncus has a slight variation from extremely tapering to trapezoidal apex in this species. Additionally, although the shape of the valva has never been cited as a diagnostic character, it appears to serve as an effective identification key distinguishing *R. erxlebella* from *R. pronubella*.

## Identification Keys

### Identification Key to the genus *Roeslerstammia*

**Table:** 

1	Distinct marking present on the fore-wing.	2
–	Distinct marking absent on the fore-wing.	3
2	The creamy-white marking only present adjacent to the costa of the fore-wing.	*R. tianpingshana*
–	The creamy-white marking adjacent to the tornus.	*R. metaplastica*
3	Phallus slightly curved, hand-shaped end of valva	*R. erxlebella*
–	Phallus approximately straight, angled at the basal one-fourth, circular-shaped end of valva	*R. pronubella*

Two species, *R. erxlebella* and *R. pronubella*, were identified, based on the presence of yellow colouration on the hind-wings (Moriuti 1972). However, this characteristic has been observed in both species (Agassiz 1996, Huemer and Tarmann 2001, Hirowatari et al. 2012). Therefore, the genital dissection is required for the accurate identification of the two species.

## Analysis

### DNA barcoding analysis

**Figure 4.**
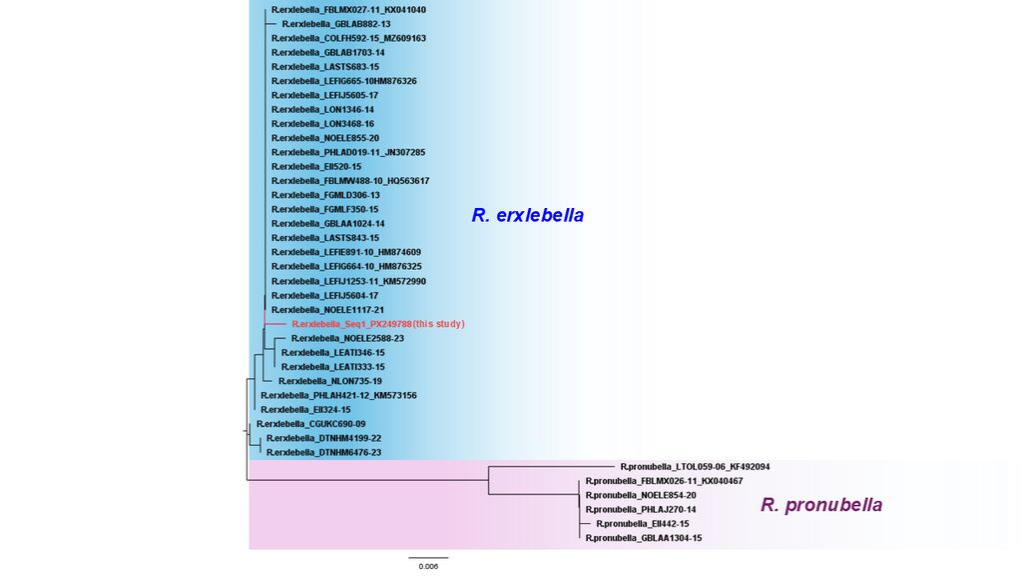
Neighbour-Joining (NJ) tree of the genus *Roeslerstammia* was calculated, based on the COI dataset including public BOLD data. The sequence name comprises scientific name_sequence ID_Genbank accession number.

The Neighbour-Joining (NJ) tree (Fig. 4) indicates that the sequence of an examined specimen is nested within the clade of the species, *Roeslerstammia erxlebella*. This analysis suggests a consistent result with morphological examination. The maximum intraspecific divergence of *R. erxlebella* is 0.82%. The minimum interspecific divergence of the most similar species, *R. pronubella*, is calculated as 5.08 - 6.15% (average: 5.54%, n=192).

## Supplementary Material

XML Treatment for Roeslerstammia erxlebella

## Figures and Tables

**Figure 1a.**
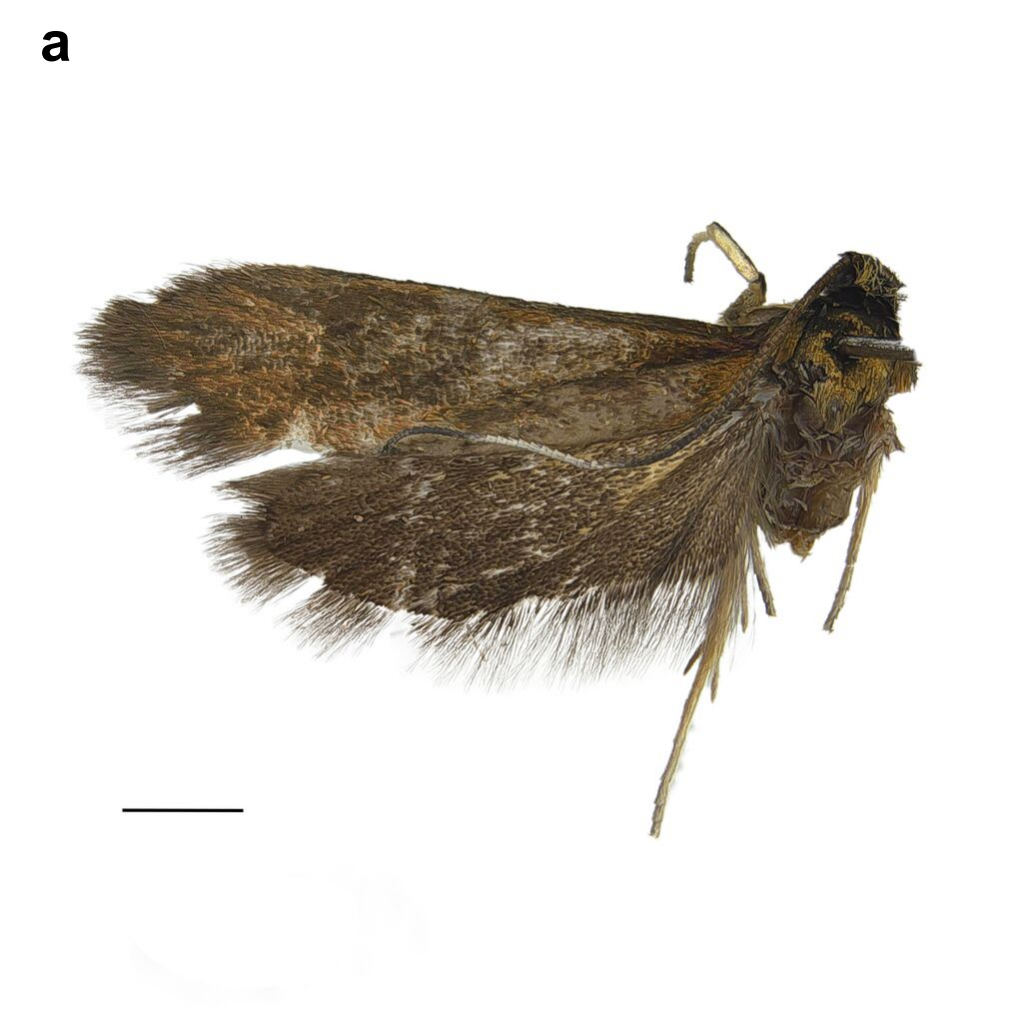
Adult;

**Figure 1b.**
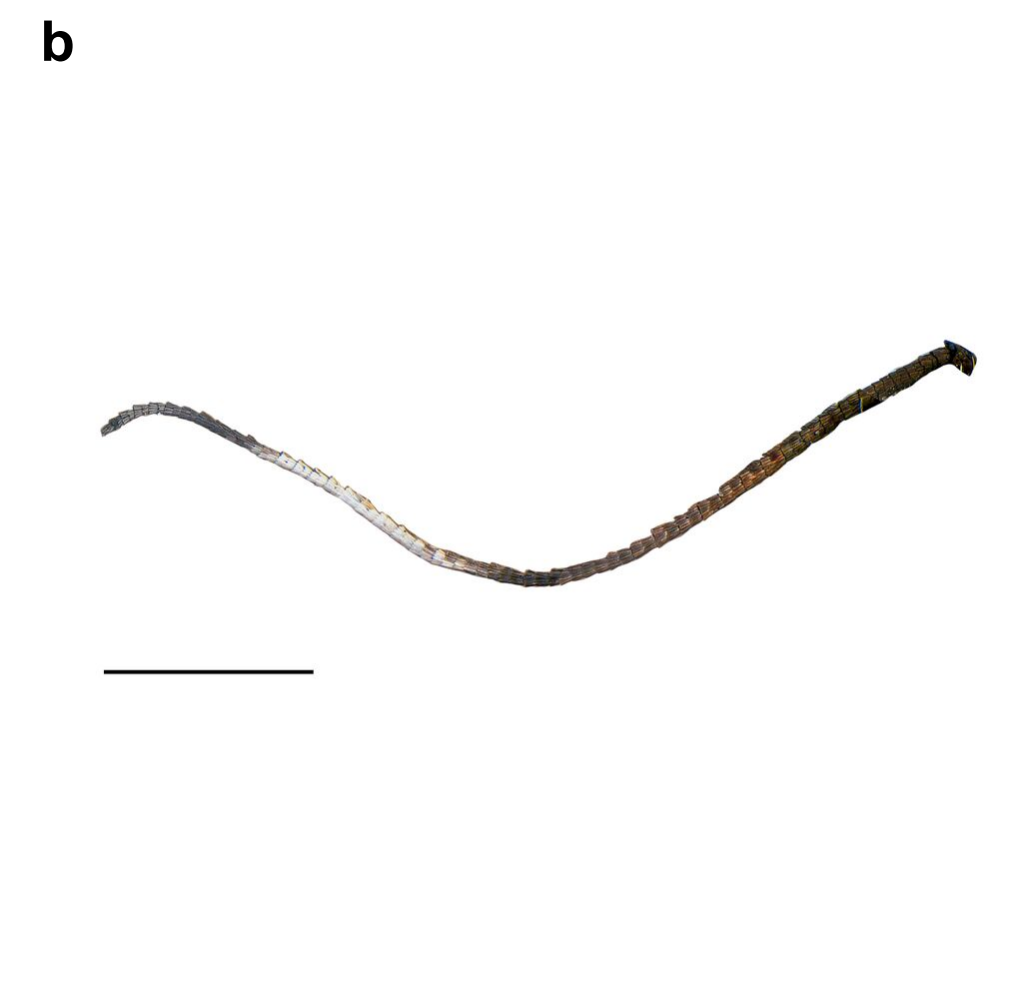
Antenna;

**Figure 1c.**
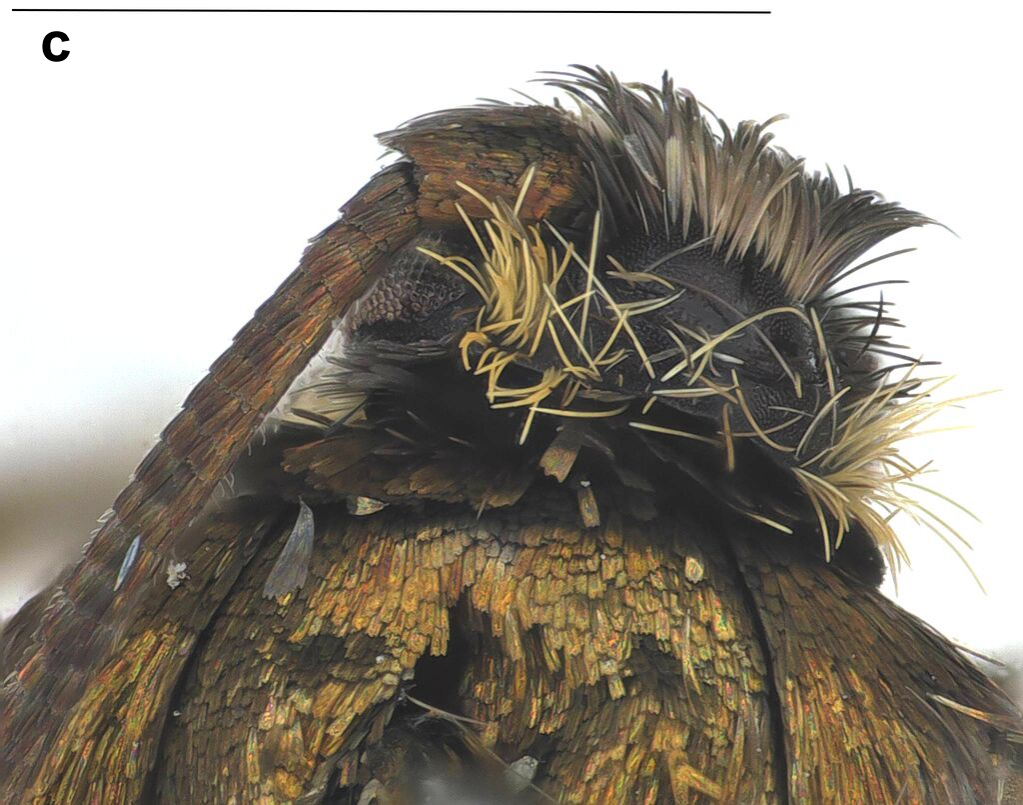
Dorsal view of head;

**Figure 1d.**
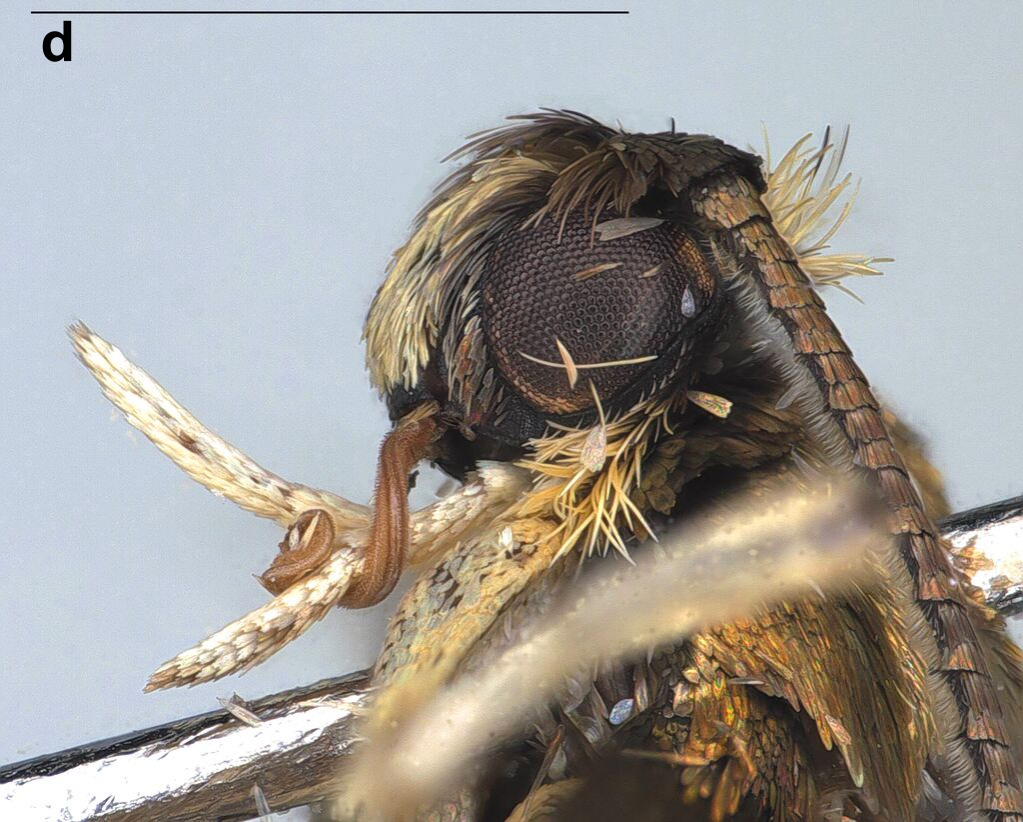
Lateral view of head.

**Figure 2a.**
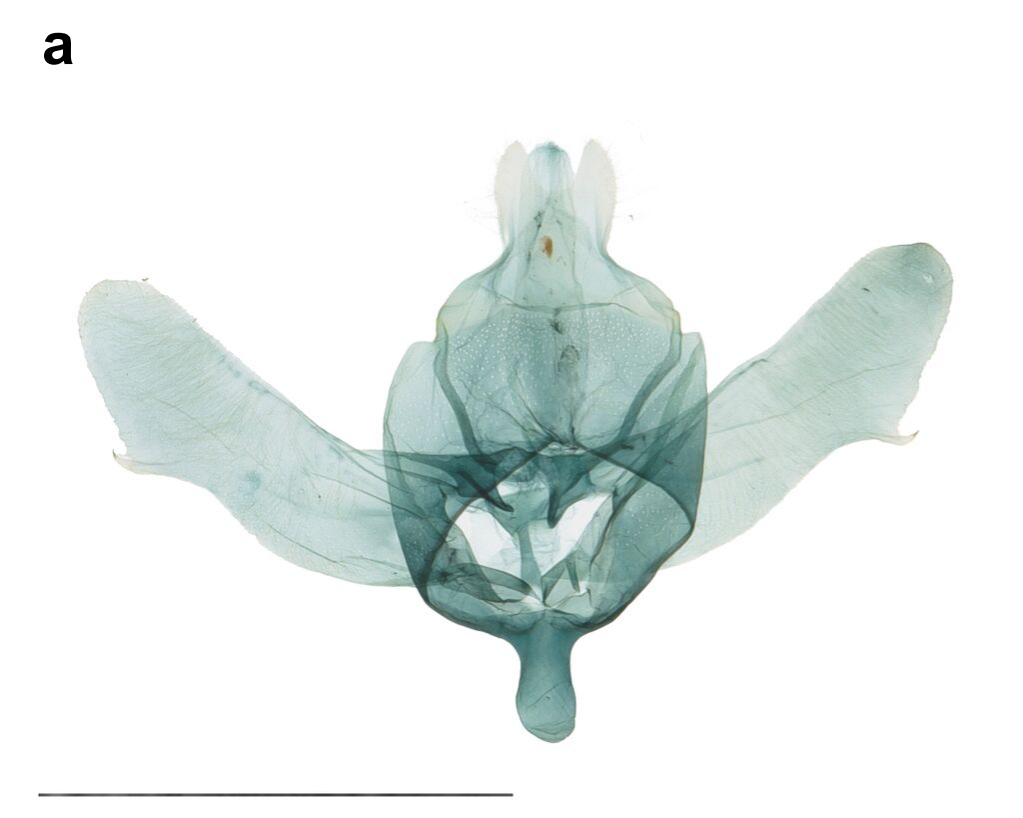
Genital capsule;

**Figure 2b.**
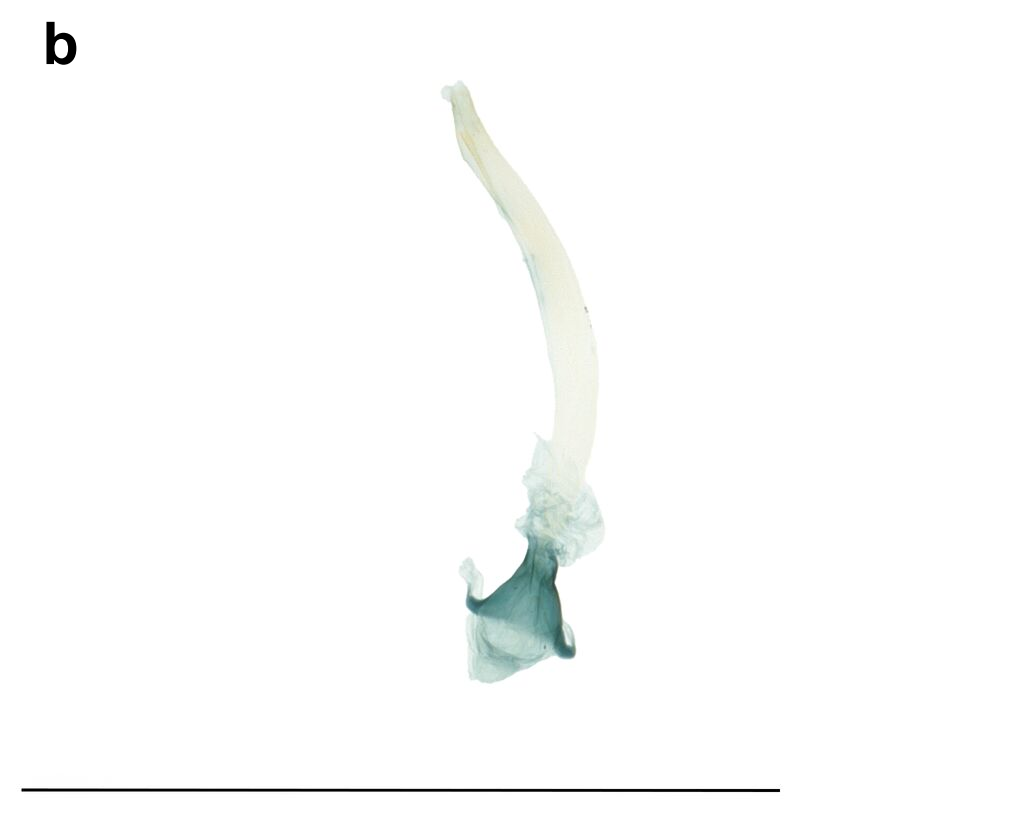
Phallus.

**Figure 3a.**
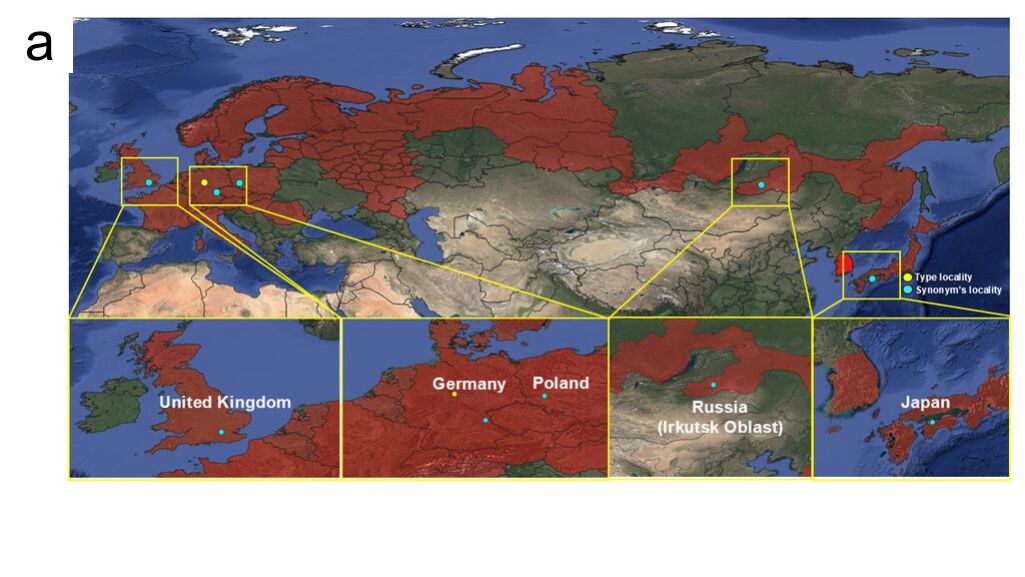
Distribution map of *Roeslerstammia erxlebella* (Fabricius 1787, Haworth 1828, Treitschke 1833, Zeller 1839, Moriuti 1972, Budashkin and Kostjuk 1993);

**Figure 3b.**
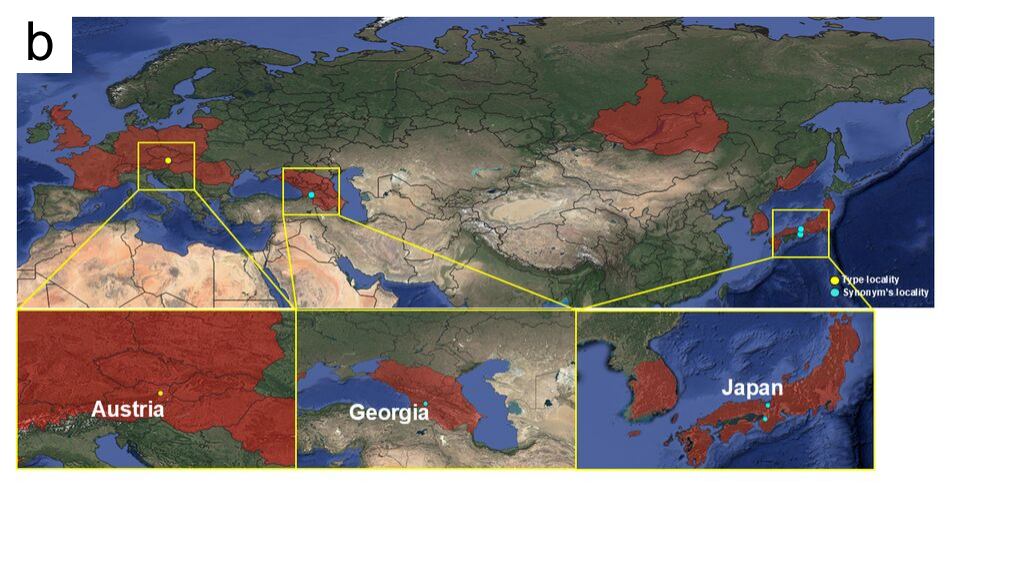
Distribution map of *Roeslerstammia prunobella* (Denis and Schiffermüller 1775, Toll 1958, Moriuti 1972);

**Figure 3c.**
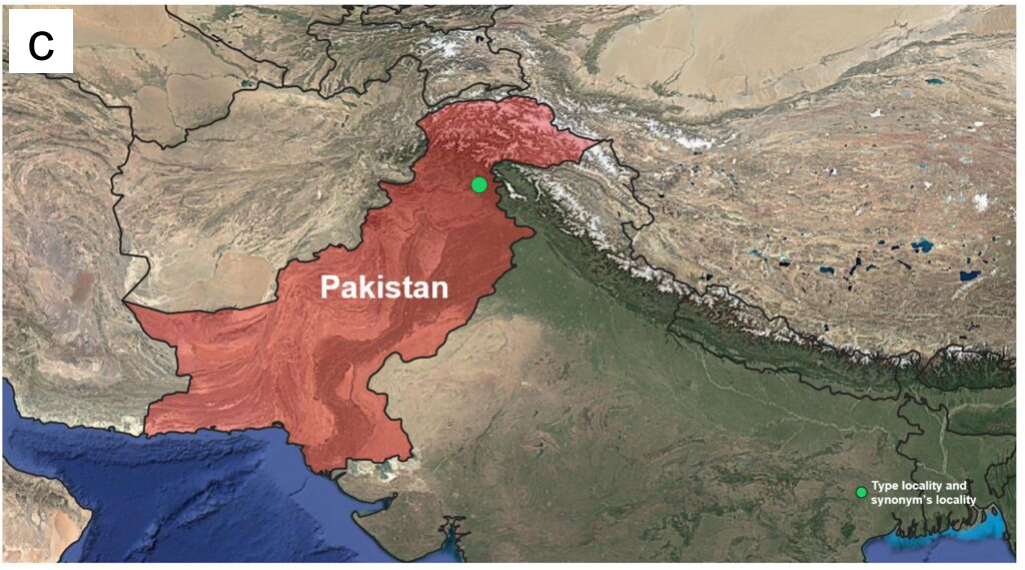
Distribution map of *Roeslerstammia metaplastica* (Meyrick 1921, Meyrick 1922). In Hirowatari's paper (Hirowatari et al. 2017), the checklist listed the type locality as 'India'. However, the actual type locality, Murree, is now located in Pakistan;

**Figure 3d.**
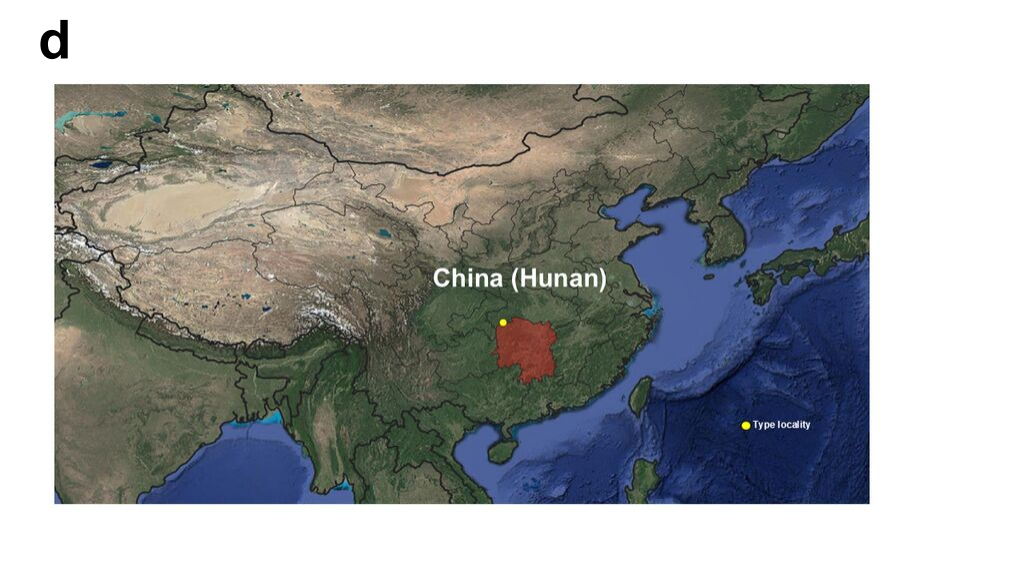
Distribution map of *Roeslerstammia tianpingshana* (Hirowatari et al. 2017).
